# Effects of Multiple Freeze/Thaw Cycles on Measurements of Potential Novel Biomarkers Associated With Adverse Pregnancy Outcomes

**DOI:** 10.16966/2572-9578.107

**Published:** 2017-02-21

**Authors:** Sandra L Rebholz, John T Melchior, Jeffrey A Welge, Alan T Remaley, W Sean Davidson, Laura A Woollett

**Affiliations:** 1 Departments of Pathology and Laboratory Medicine, Ohio, USA; 2 Psychiatry and Behavioral Neuroscience, Ohio, USA; 3 University of Cincinnati Medical School, Cincinnati, Ohio; Lipoprotein Metabolism Section, Cardio-Pulmonary Branch, National Heart, Lung, and Blood Institute, National Institutes of Health, Bethesda, Maryland, USA

**Keywords:** Cholesterol, Cytokines, Lipoprotein sizing, HDL proteome, Apolipoprotein A-I, preterm, low birthweight

## Abstract

World-wide, millions of women enter preterm labor or have small newborns. Effective biomarkers are needed to identify women at risk for these adverse outcomes. A time and cost effective way to examine any potentially new biomarkers in samples collected during prior studies or trials that had been assayed for other metabolites would be highly useful. Thus, the current study aimed to determine if samples that had been previously thawed and re-frozen could be re-assayed for novel biomarkers, those being lipoprotein composition (sizing, proteome, lipids) and combined cholesterol and cytokine concentrations. Fasting blood was collected from 51 young non-pregnant women and plasma was analyzed for lipoprotein composition and cytokine concentrations after multiple freeze/thaw cycles in the cold or at room temperature and after being stored for 18 months. Plasma LDL-C, HDL-C, total cholesterol, and triglyceride concentrations decreased <6–7% (cholesterols) or <20% (triglyceride) after 7 thaws in the cold, 3 thaws at room temperature, and after 18 months of storage. As these decreases were less than day-to-day reported variation of lipids, they do not appear to be physiologically significant. Cytokine (IL-6, TNF α, IL-8, IL-1β) and hsCRP concentrations decreased by 22%, 8%, 8%, 22%, and 35%, respectively; only IL-6, IL-1β and hsCRP concentrations showed significant decreases greater than day-to-day variations of 20%. For measured triglyceride and cytokine, but not cholesterol concentrations, decreases with freeze/thaw cycles were greater when concentrations were elevated. Multiple thaws also led to changes in lipoprotein sizing, specifically to a shift from medium- and large-sized HDL particles to small-sized HDL particles and from large LDL to IDL. No changes occurred for VLDL particle numbers. Though particle sizes changed, the HDL proteome did not change with multiple thaw cycles or after long term storage. Overall, the results demonstrate that it is possible to use previously obtained frozen samples for plasma cholesterol and triglyceride levels and the lipoprotein proteome, and lipoprotein sizing and cytokine concentrations if one knows the history of the sample as changes should be relative to one another.

## Introduction

New biomarkers are needed to identify women at risk for adverse pregnancy outcomes, including those having infants with low birth weight (LBW) or that are born preterm (preterm birth; PTB). In fact, world-wide there are greater than 22 million LBW and 15 million PTB infants born each year [1,2]. Preemptive identification of women at risk of adverse pregnancy outcomes would allow for women at high risk to be targeted for monitoring or interventions to improve outcomes. This is especially true in resource-poor settings where a majority of the infants with LBW or PTB are born [1,2] and where medical help is not readily available. While there are known mediators of fetal growth (nutrition, placental function) and preterm birth (cervix length, previous preterm birth, spacing between pregnancies), there are many newborns with idiopathic causes of LBW or PTB. In fact, in the past five years, there are over 800 articles in Pub Med evaluating potential biomarkers for preterm birth and over 350 articles evaluating potential biomarkers for low birth weight infants.

Though a number of previous studies have examined cytokines in the maternal circulation as potential biomarkers for preterm birth or low birth weight infants, less than 50 publications have presented maternal lipid levels and only one has examined lipoprotein size or the lipoprotein proteome, the best current indicators of metabolic lipid disorders and disease states [3–5]. It is surprising that more studies have not looked for a potential relationship between cholesterol and adverse pregnancy outcomes because cholesterol is a precursor for estradiol and progesterone, is a key component of every membrane, and is concentrated in parts of the membrane where intracellular signaling often originates. Likewise, lipoproteins are the route by which lipids, such as cholesterol, and a number of metabolically active proteins, such as those with anti-inflammatory functions, are transported between tissues. Of the few that have examined maternal cholesterol and adverse pregnancy outcomes, associations include positive, negative, or null. Thus, it may not be a simple measurement of maternal cholesterol or lipid levels, but the lipoprotein composition or the combination of various factors that contribute to the adverse outcomes. For example, it has been just recently hypothesized that maternal lipoprotein-cholesterol and cytokine concentrations (low HDL-C and high TNFα concentrations) are a biomarker for preterm birth only when combined [6].

The ability to assay previously collected samples would be a very time and cost effective way to test if any newly discovered biomarkers are associated with adverse pregnancy outcomes. As the lipoprotein proteome is a relatively recent marker for lipoprotein function and disease processes, no studies have determined if lipoprotein composition (sizing, the proteome, lipid concentrations) changes with multiple freeze/thaw cycles. Likewise, the impact of freeze/thaw using the current Multiplex systems and under the same conditions used to study lipoprotein composition after several freeze/thaw cycles are unknown. Thus, the goal of this study was to determine if samples previously collected and analyzed for various metabolic factors can be re-assayed for lipoprotein composition and/or cytokines. Results obtained can be applied to any studies interested in lipoprotein composition and/or cytokine levels, however, and not just studies focused in adverse pregnancy outcomes.

## Research design and Methods

### Human participants

**Figure 1:**
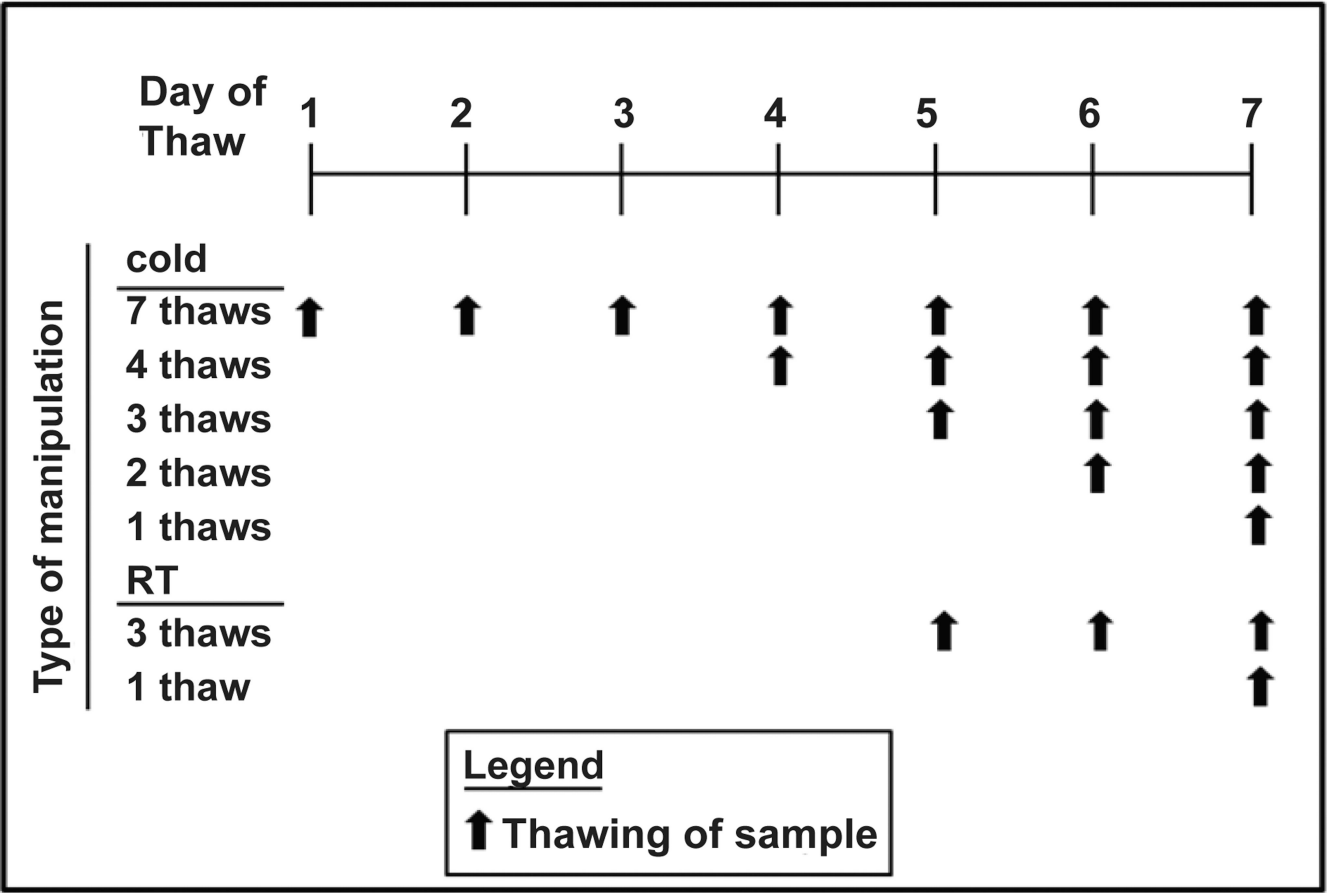
Scheme for thawing and refreezing samples at room temperature or in the cold. The final thaw was under the conditions of each typical assay.

Up to 30 ml of blood was collected from 51 healthy non-pregnant young women, aged 18–44 years old, at the Clinical Translational Research Center (CTRC) at Cincinnati Children’s Hospital Medical Center (CCHMC). Blood was collected after an overnight fast and plasma separated immediately. Plasma was aliquoted so that there was a separate tube for each assay and for every thaw (see Figure 1). For the lipoprotein sizing and the HDL proteome, plasma from 8 women were combined to obtain 6 samples. All samples were placed in −80°C freezer. Samples thawed in the cold were placed in racks on a tube rotator in the cold room until samples were thawed; it took at 2–3 hr for samples to thaw. Samples thawed at room temperatures were placed in a room temperature water bath until thawed, which took less than 30 min. Once fully thawed, samples were placed back in the freezer, until the next thaw. The final thaw occurred in the manner typical of the assay being run. Thus, there were up to 7 thaws for samples thawed in the cold and 3 thaws for samples thawed at room temperature. This regime was used as plasma to be used for metabolites is often thawed in the cold or on ice, but sometimes at room temperature for rapid thaws. These samples were assayed within 2–5 months of collection. One set of tubes was left in the freezer for 18 months prior to analysis. The collection and use of samples were approved by the Institutional Review Board (IRB) at the University of Cincinnati.

### Plasma lipoprotein-cholesterol and triglyceride concentrations

Plasma was analyzed for LDL-cholesterol (LDL-C), HDL-cholesterol (HDL-C), total cholesterol, and triglyceride levels using a Roche reagent on a Roche/Hitachi Cobas c systems auto analyzer (Roche Diagnostics, Indianapolis, IN). LDL- and HDL-C were directly measured using second and third generation assays [7], respectively. Assays were run in the Biochemistry Core at CCHMC.

### Plasma cytokine concentrations

Cytokine (IL-1β, IL-6, IL-8,TNFα) concentrations in the sample supernatants were determined by enzyme-linked immunosorbent assay (ELISA) using Milliplex™ Multiplex kits (Millipore, Billerica, MA). Assays were run in the Cytology Core at CCHMC. Samples with values below the detection limit were assigned levels of 0.03 mg/L (halfway between the lowest detection limit and 0).

### Plasma hsCRP concentrations

hsCRP concentrations in the sample supernatants were determined by ultra sensitive enzyme-linked immunosorbent assay (ELISA) from Cal biotech (Cal biotech, El Cajon, CA). Assays were run in the Cytology Core at CCHMC. Samples with values too high were assigned levels of 40 mg/L and those less than detection were assigned levels 0.03 mg/L.

### Plasma apoA-I concentrations

Samples were assayed for dpolipoprotein A-I (dpo A-I) using an ELISA assay produced by Mabtech. Any sample with values >4.5 mg/ml was not included in the analysis as this value is routinely re-run; samples from 9 participants were not used in the analyses.

### Lipoprotein sizing

Samples of the lipoprotein particle analysis by proton NMR spectroscopy were shipped on dry ice to NIH. Particle concentrations of lipoproteins of different sizes were calculated from the measured amplitudes of their spectroscopically distinct lipid methyl group NMR signals [8,9]. Weighted-average lipoprotein particle sizes are derived from the sum of the diameter of each subclass multiplied by its relative mass percentage based on the amplitude of its methyl NMR signal.

### HDL proteomics

Frozen plasma was thawed, and non-apoB lipid-containing particles were isolated and consisted of primarily HDL; HDL was not isolated by ultracentrifugation because of known losses in the HDL proteome during ultracentrifugation [10]. Proteins devoid of lipids were separated from the lipid-bound proteins with calcium silicate hydrate (lipid removal agent, LRA) [11]. Lipid-bound proteins were digested overnight with trypsin and prepared for mass spectrometry as described [12].

### Statistics

Data are presented as individual measurements for each participant for lipid, apoA-I, and cytokine concentrations. Plasma was pooled for some of the assays and each pooled sample is presented for the lipoprotein sizing and HDL proteomics. To achieve normality and allow for convenient ratio interpretation of effects, data were analyzed by repeated-measures ANOVA on log-transformed values, and results are back-transformed to the original scale (i.e., effects of repeated thaws are expressed as estimates of ratios and their confidence intervals). All values were compared to the first thaw in the cold except for the 18 month samples. As these samples were assayed using different reagents we compared these data to those obtained in the first thaw by paired t-tests after log-transforming values. We did not employ conventional null hypothesis tests (of whether ratios are equal to one) for two reasons. First, some endpoints showed very little intra-individual variation (small coefficient of variation), so differences between conditions could be shown to be non-null yet small enough to be scientifically insignificant, as defined below. Conversely, where variation was substantial, ratios had wide confidence intervals, such that neither null effects nor substantial effects could be ruled out (possible Type II error). To mitigate against these issues, we deemed differences for cholesterol, LDL-C, and HDL-C concentrations greater than 6%, 7%, or 6% or differences for triglyceride or cytokine concentrations greater than 20% as scientifically significant [13–15]. For the pooled samples in which there were multiple measurements of sizing or the proteome, we used conventional statistics that were initially unadjusted for the number of proteins analyzed analyzed and then adjusted for multiple testing using the Bonferroni correction, and were not log-transformed. All averaged data are presented as means ± SD.

## Results

**Figure 2:**
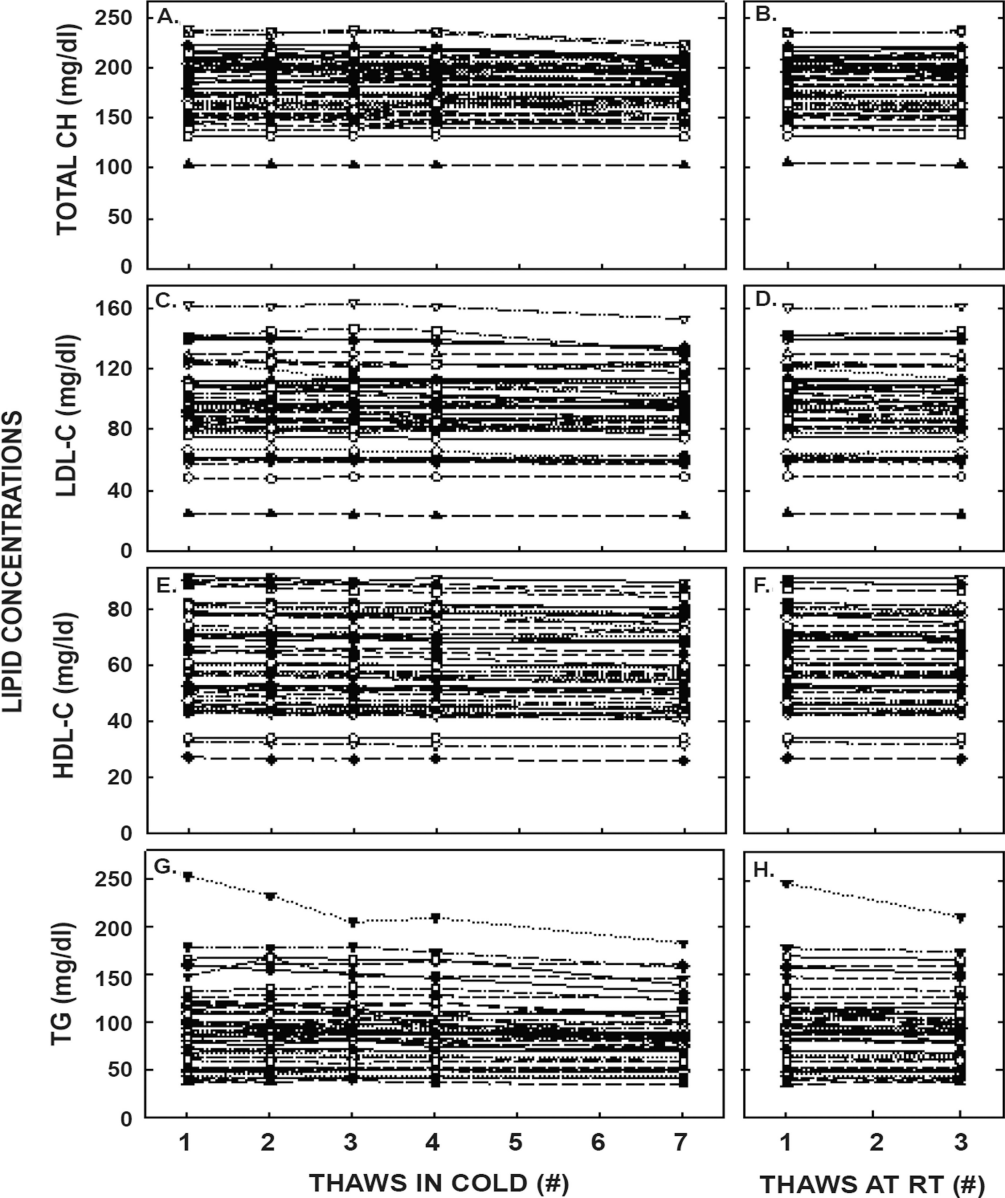
Plasma lipid concentrations after multiple thaws in the cold (left panels) or at room temperature (right panels). Fasted blood samples were collected from 51 healthy young women aged 18–44 years old. Total cholesterol (A, B), LDL-C (C, D), HDL-C (E, F), and triglyceride (G, H) concentrations were measured in each of the aliquots thawed 1, 2, 3, 4 or 7 times in the cold and 1 or 3 times at room temperature. Each point represents an individual participant.

Plasma triglyceride and lipoprotein-cholesterol concentrations for each thawed sample from every participant are shown in Figure 2 and averaged values are found in Table 1. Interestingly, statistically significant differences occurred as soon as the second thaw for HDL-C concentration with a decrease of 0.3 mg/dl in concentration, the third thaw for LDL-C (0.7 mg/dl decrease) and total cholesterol concentrations (1.1 mg/dl decrease), and the fourth thaw for triglyceride concentration (3.1 mg/dl). Thawing at room temperature had little effect when compared to thawing in the cold. The greatest effects occurred after 7 thaws in the cold and included percent decreases of 3.5 ± 0.5, 2.2 ± 0.2, 4.5 ± 0.7 and 8.6 ± 1.1% for LDL-C, HDL-C, total cholesterol, and triglyceride concentrations, respectively. After being stored for 18 months, concentrations of all lipids were not different from values of samples that had only been thawed the original time. The reductions in measured concentrations from the first to seventh thaw were similar for the participants with the 10 highest and 10 lowest values for LDL-C, HDL-C, and total cholesterol (all comparisons were within 4%). However, there was a greater decrease (14.7%) of plasma triglyceride concentrations for the 10 highest values versus the 10 lowest values (0.4%).

**Figure 3:**
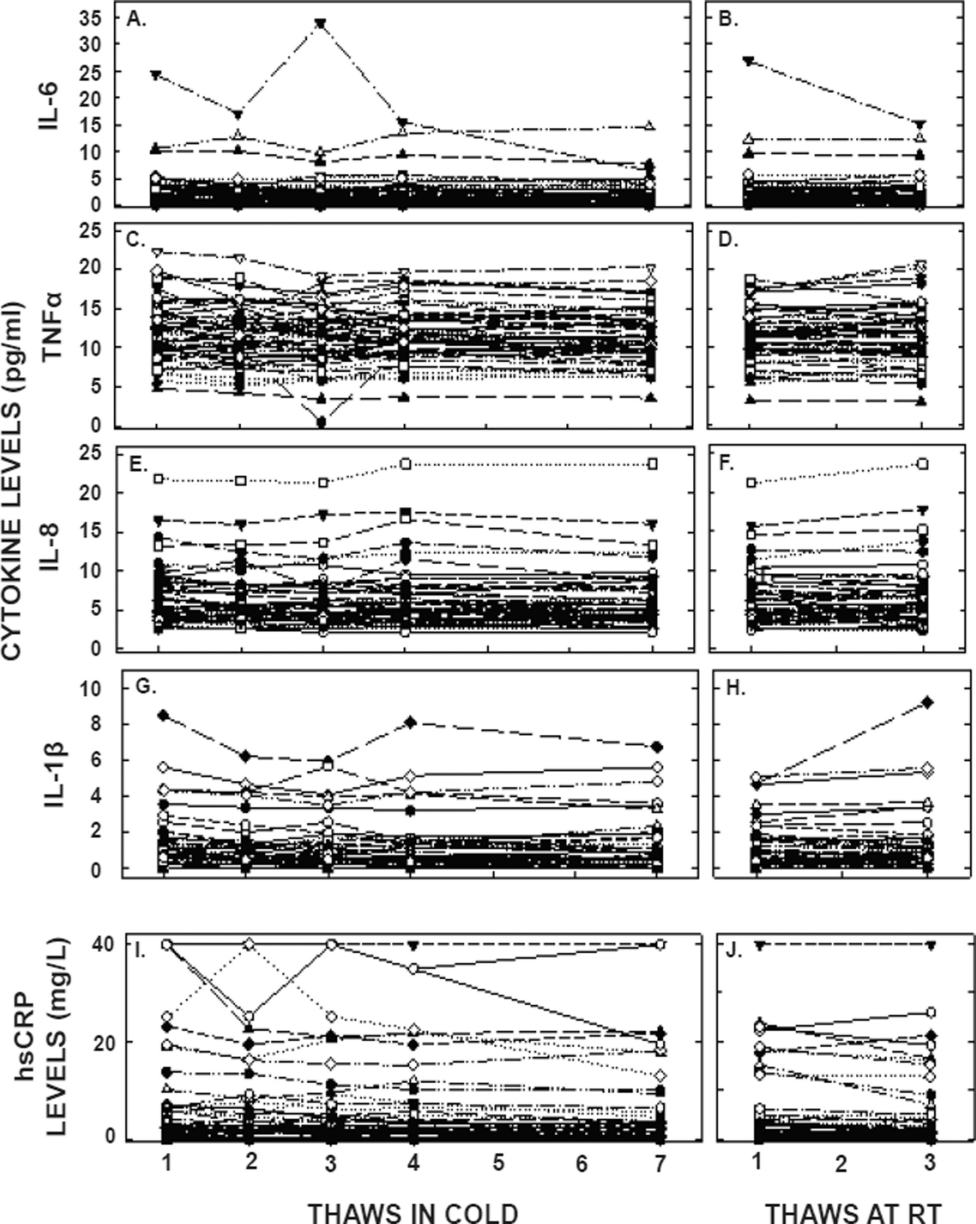
Plasma cytokine concentrations after multiple thaws in the cold (left panels) or at room temperature (right panels). Samples were the same as those in Figure 1. hsCRP (A, B), TNFα (C,D), IL-6 (E, F), IL-8 (G, H), and IL-1β (I, J) were measured in each of the aliquots thawed up to 7 times in the cold or 3 times at room temperature. Each point represents an individual participant. It should be noted that there were two participants with excessively high values of some of the cytokines and are not on the graph; concentrations for IL-1β not shown were 49, 23, 82, 32 and 18 pg/ml(participant A) and 117, 136, 76, 106, and 108 pg/ml (participant B), for IL-6 were 24, 17, 32, 16, and 6 pg/ml (A), for TNFα were 74, 45, 56, 48 and 26 pg/ml (A) and 88, 64, 52, 73, and 81 pg/ml (B).

Plasma cytokine and hsCRP measurements were also changed significantly with freeze/thaw cycles (Figure 3 and Table 1) and to a greater extent than that for lipids. There were significant decreases in concentrations of all inflammatory markers with freeze/thaw cycles, with less of an effect when thaws were at room temperature. While the concentrations of TNF α and IL-8 changed less than a day-to-day variation of 20%, and were 8% and 8% respectively by the seventh thaw, the concentrations for IL-6, IL-1β and hsCRP decreased 22, 22, and 35% by the seventh thaw, respectively. After 18 months of storage, IL-8 and TNF α concentrations changed little whereas IL-6 and IL-1β decreased markedly. Unlike other factors, measured hsCRP concentrations increased after being stored for 18 months. Even though the subjects appeared to be healthy, there were 5 participants with elevated hsCRP that were too high to measure and thus concentrations were set at 40 mg/L and two subjects with IL-1β, IL-6, TNF α, and IL-8 measured values excessively elevated in the initial thaw. When we removed the hsCRP values from the averages, new averages were 3.8 ± 5.4, 3.4 ± 4.8, 3.5 ± 5.1, 3.4 ± 5.0, 3.2 ± 4.9, 3.4 ± 5.1, and 2.6 ± 4.5 pg/ml for the 5 cold and two room temperature thaws respectively. When the two participants with the highest values for cytokines were removed, new averages for IL-6 were 2.2 ± 2.2, 2.0 ± 2.4, 1.9 ± 2.0, 2.1 ± 2.4, 1.9 ± 2.4, 2.1 ± 2.3, and 2.2 ± 2.3 pg/ml, for TNF α were 12.0 ± 3.7, 11.6 ± 3.7, 11.8 ± 3.7, 11.4 ± 3.3, 11.3 ± 3.4, and 11.5 ± 3.8, for IL-8 were 6.4 ± 3.4, 6.2 ± 3.4, 6.1 ± 3.4, 6.5 ± 3.8, 6.2 ± 3.6, 6.2 ± 3.4, and 6.6 ± 3.7 pg/ml, and for IL-1β were 1.3 ± 1.7, 1.2 ± 1.4, 1.1 ± 1.5, 1.2 ± 1.6, 1.1 ± 1.6, 1.1 ± 1.3, and 1.2 ± 1.8 pg/ml, for the 5 cold and two room temperature thaws, respectively.

**Figure 4:**
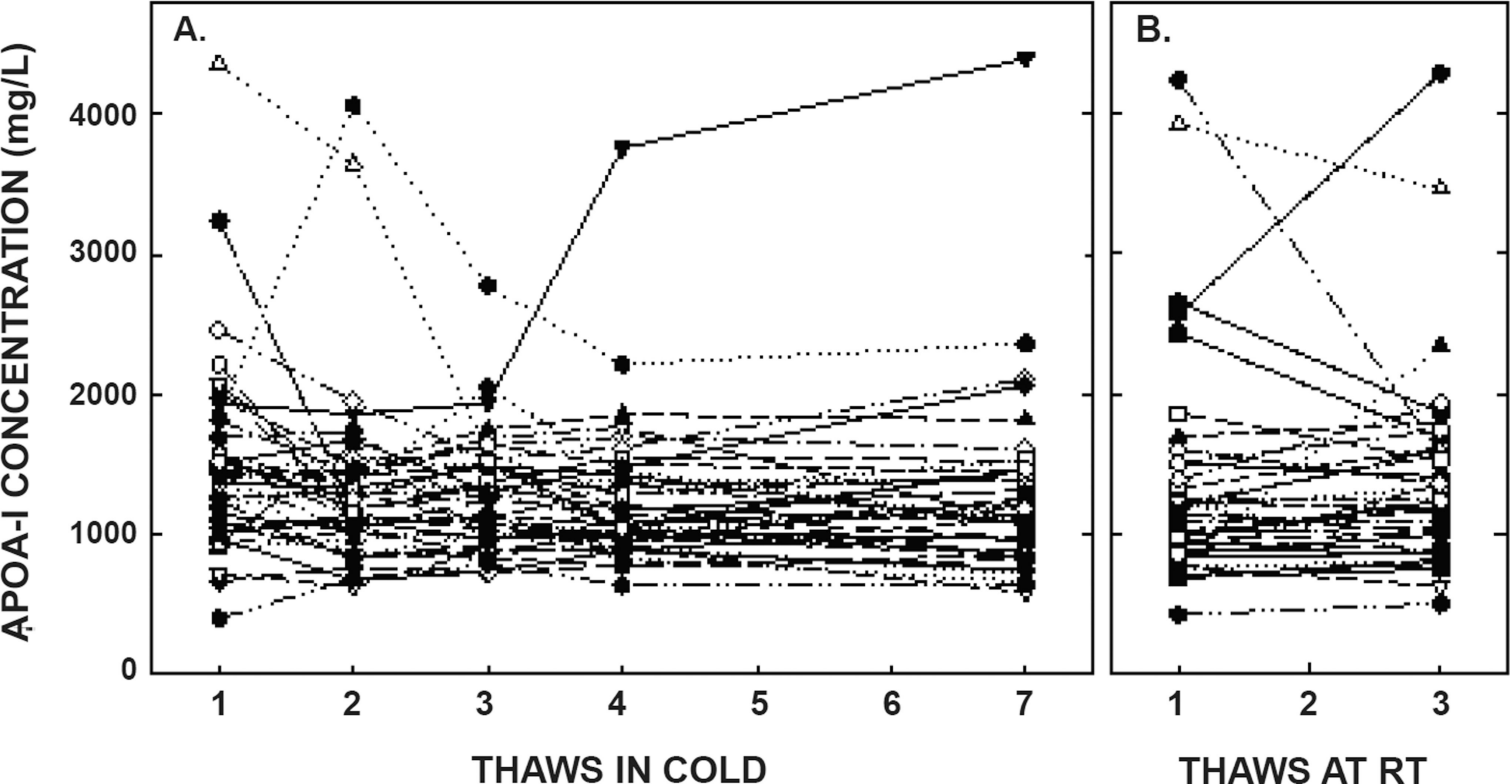
ApoA- I concentrations after multiple thaws in the cold (left panel) or at room temperature (right panel). Samples were the same as those described in Figure 1. ApoA-I was measured in each of the aliquots thawed up to 7 times in the cold or 3 times at room temperature. Each point represents an individual participant.

**Figure 5:**
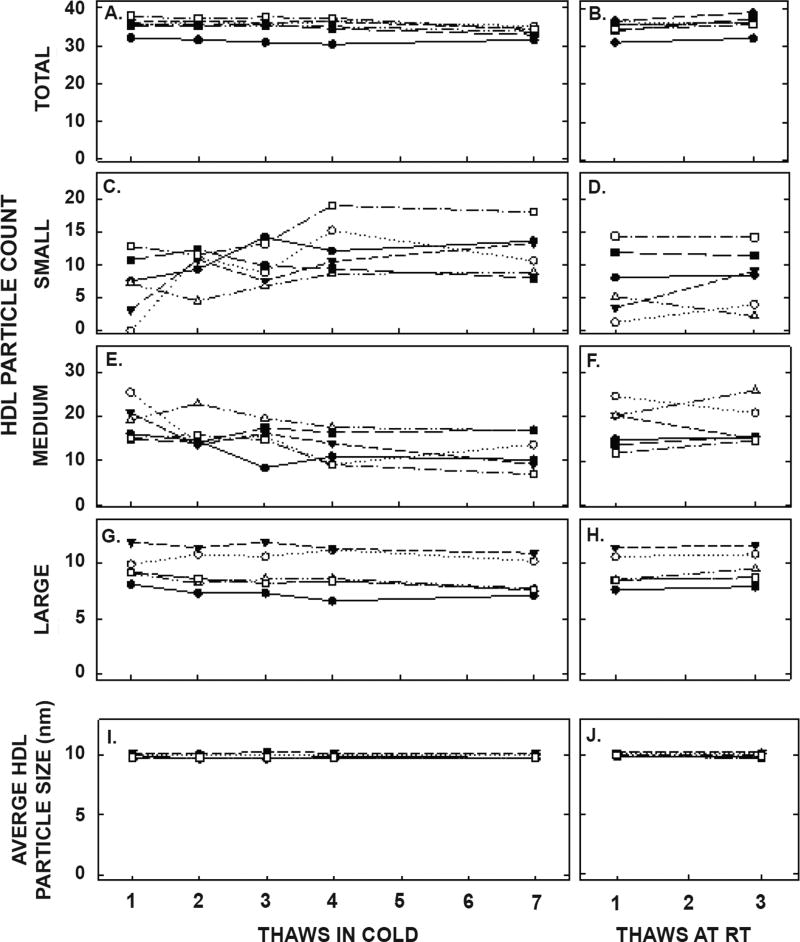
HDL particle counts and average HDL size in samples described in Figure 1 thawed multiple times in the cold (left panels) or at room temperature (right panels). Each data point represents plasma from participants described in Figure 1 that were pooled (8 samples pooled).Total HDL particle count (A, B) and particle counts of small HDL (C, D), medium HDL (E, F), and large HDL (G, H) were measured by NMR. The average size of HDL particles were also determined (I, J).

**Figure 6:**
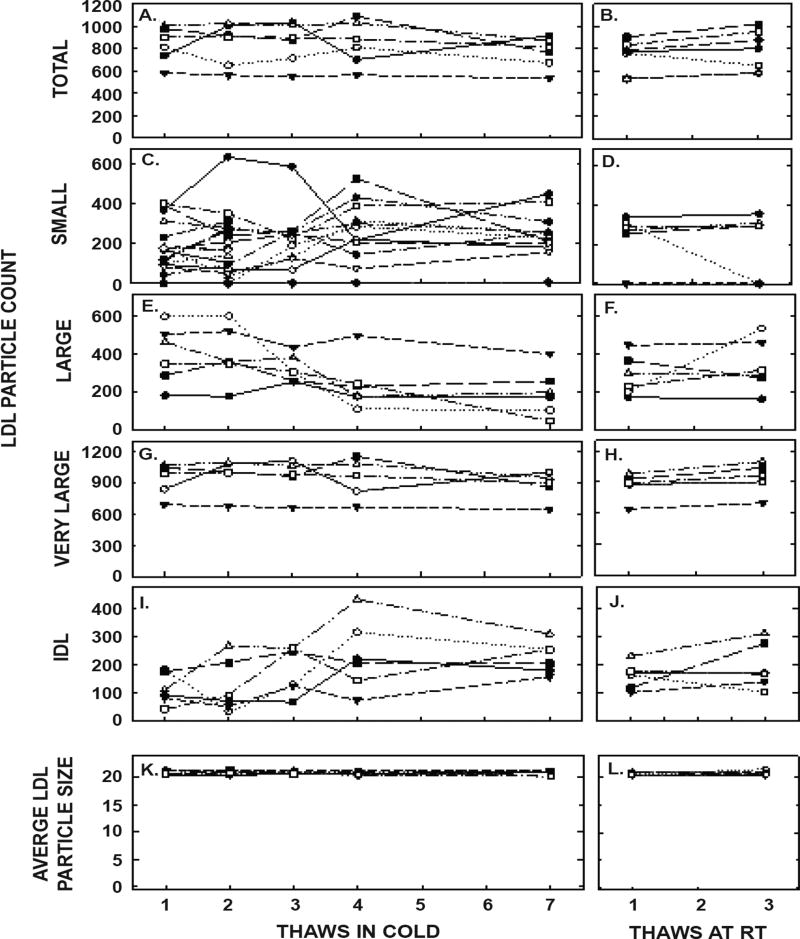
LDL particle counts and average LDL size in pooled samples described in Figure 5 thawed multiple times in the cold (left panels) or at room temperature (right panels). Total LDL particle count (A, B) and particle counts of small LDL (C, D), large LDL (E, F), very large LDL (G, H), and IDL were measured by NMR. The average sizes of LDL particles were also measured (I, J).

**Figure 7:**
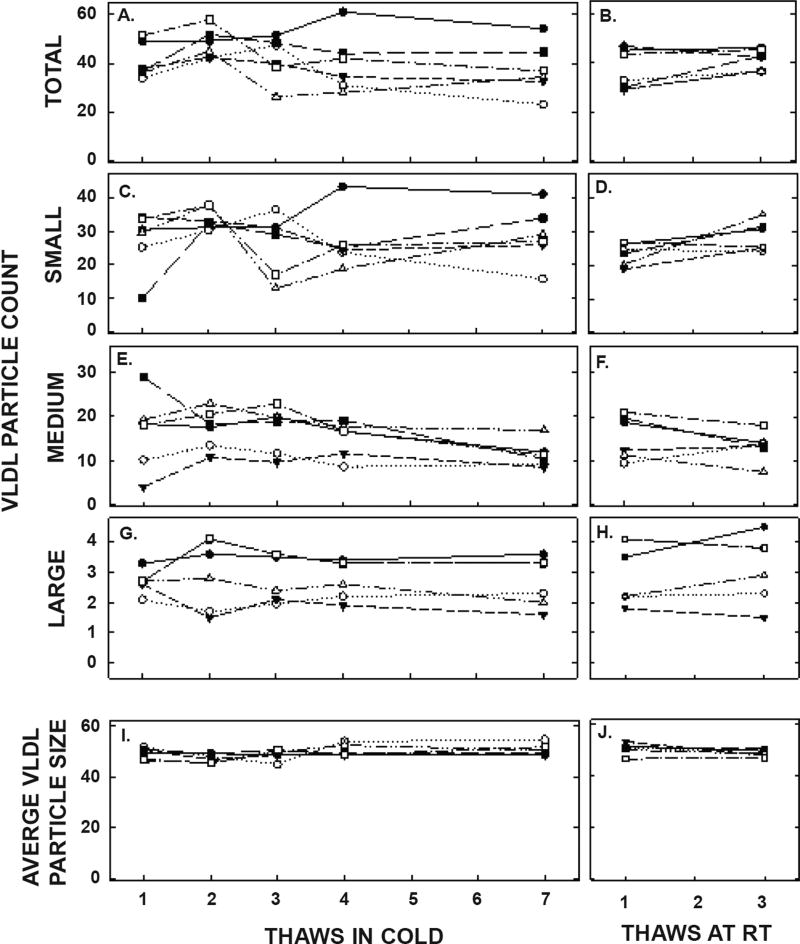
VLDL particle counts and average VLDL size in pooled samples described in Figure 5 thawed multiple times in the cold (left panels) or at room temperature (right panels). Total VLDL particle count (A, B) and particle counts of small VLDL (C, D), medium VLDL (E, F), and large VLDL (G, H) were measured by NMR. The average sizes of VLDL particles were also measured (I, J). Each data point represents plasma from participants described in Figure 6.

ApoA-I concentrations are presented in Figure 4 and Table 1. As with the other parameters, there was a statistically significant decrease in concentrations that occurred with multiple thaws in the cold (13% decrease) but little change with three thaws at room temperature. After 18 months of storage, apoA-I concentrations increased 40%, with the greatest increases occurring in the samples with the highest concentrations originally. Possibly the most significant physiological change that occurred with the multiple thaws was a shift in the size of lipoproteins. One or two thaws had little impact on lipoprotein sizing. However, after 3 freeze/thaws, there was a decrease in the number of medium and large HDL particles associated with a near doubling in the number of small HDL particles (Figure 5 and Table 2). Interestingly, three thaws at room temperature had little effect on sizing, nor did long term storage. LDL particle size also shifted with a decrease in large LDL and an increase in IDL (Figure 6 and Table 2). There was little effect of freeze/thaw cycles on sizes of VLDL particles (Figure 7 and Table 2).

**Figure 8:**
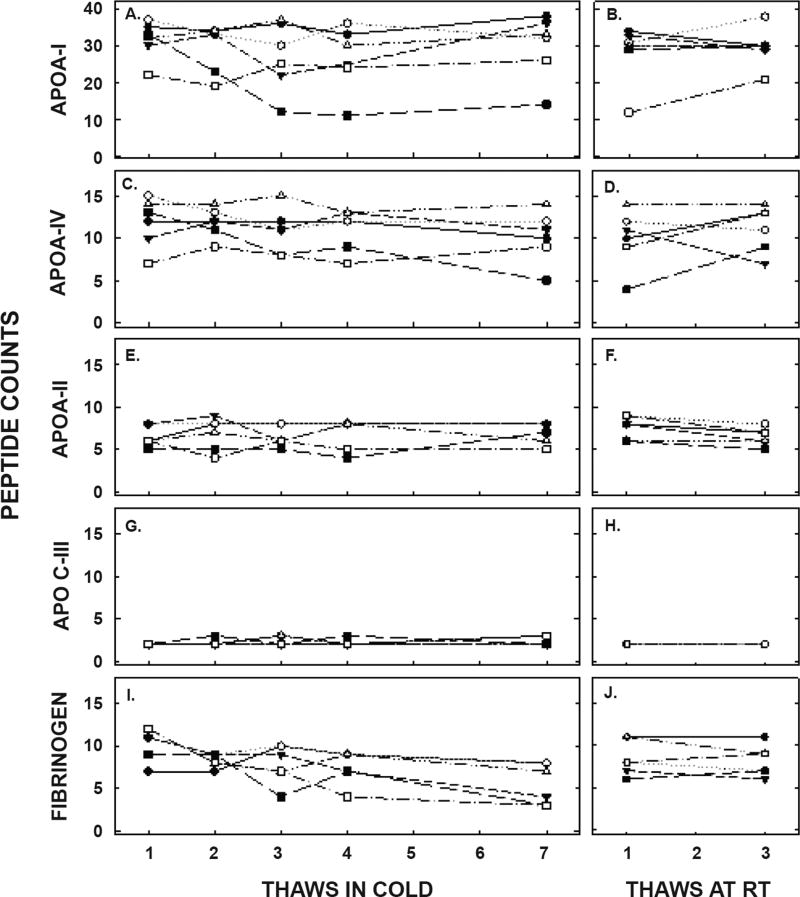
Peptide counts of proteins associated with HDL that are related to lipid metabolism (apolipoproteins) or were changed with freeze/thaw cycles (fibrinogen); up to 86 different proteins were detected on the HDL particles of different participants. Peptide counts for apoA-I (A, B), apoA-IV (C, D), apoA-II (E, F), apoC-III (G,H), and fibrinogen (I, J) were measured by mass spec in samples thawed up to 7 times in the cold (left) and up to 3 times at room temperature (right). Each data point represents pooled plasma from participants as described in Figure 5.

Finally, the proteome of apo B-depleted lipid-containing particles, made up primarily of HDL, was determined by mass spec and showed a total of 86 different peptides. We analyzed the top 40 peptides, as those proteins were consistently present in all samples. The peptide counts for the most abundant apolipoproteins are presented in Figure 8 and Table 3. ApoA-I was the apolipoprotein with the greatest number of peptide counts, and was second in abundance to complement C3; albumin was also present in the samples due the high levels of albumin in blood. There was no effect of thawing and refreezing on the relative amount of apoA-I or on other apolipoproteins, including apoA-IV, apoA-II, and apoC-III. Of the 40 peptides with the greatest counts, only 2 were modestly reduced with several thaws, fibrinogen α chain and fibrinogen β chain, when unadjusted for the number of comparisons made (10.0 ± 2.0 to 6.0 ± 2.4 and 8.8 ± 1.0 to 5.0 ± 1.9 between the first and seventh thaw, respectively). When adjusted for the number of comparisons, there were no effects of thawing on the proteome.

## Discussion

A large number of studies have examined the ability of various metabolites to be used as biomarkers for adverse pregnancy outcomes. The ability to go back and analyze these stored samples for new potential biomarkers for LBW or PTB infants, or other disease states would be very time and cost effective and could lead to a quicker intervention or method of detection. The aim of the current study was to determine if samples thawed a number of times could still yield results that are physiologically relevant when novel biomarkers are assayed. This is the first report to show effects of freeze/thaw cycles on novel biomarkers lipoprotein sizing and the lipoprotein proteome. This is not the first report to study the effect of freeze/thaw cycles on concentrations of plasma lipids or cytokines, and there are even reports summarizing the data from previous studies [16– 21]. However, none of the previous studies compared the impacts of many thaws on lipid and inflammatory factor concentrations in both the cold and at room temperature. Many did not evaluate the current generation of assays as well.

Unlike previous studies that showed no effects of freeze/thaw cycles on plasma cholesterol and triglyceride concentrations, we found statistically significant decreases in concentrations of all plasma lipids with freeze/ thaw cycles due to the high precision of the repeated measures of 51 participants. This is apparent as some of the significant decreases occurred as early as the second thaw and with decreases of merely 1%. As there have been reports of as little as 6%, 7%, and 6% day-to-day variation of plasma cholesterol, LDL-C and HDL-C levels, respectively [13–15], a reduction of concentration with as little as 1% is less that the day-to-day variation. Measurements less than the day-to-day variation, which accounted for lipid concentrations after all manipulations, were thus considered non-significant. Even triglyceride concentrations, which decreased 10% by the seventh thaw, were still less than the day-to-day variation reported for triglycerides (20%), and was considered a non-physiological decrease as well [13–15]. Even though there was a statistical decrease, the same percent change occurred for values that were the greatest and the least LDL-C, HDL-C and total cholesterol concentrations. In contrast, there was a greater decrease in measured triglyceride concentrations for the greatest *vs* least values.

Due to recent studies which suggest that lipoprotein-cholesterol concentrations are not really indicative of lipoprotein metabolism and dysregulation, lipoprotein size and proteome were also measured. Though sizing of lipoproteins is only a snapshot of metabolism, it is a better indicator of altered metabolism. For example, small HDL particles are thought to be best at effluxing cholesterol out of cells whereas larger HDL may be taken up more readily by tissues. While the ability of take up cholesterol from cells was always thought to be the key function of HDL, it is now thought that HDL could deliver various factors to tissues as HDL particles carry >90 proteins and >100 different lipids, many of which are bioactive [4, 5,22–24]. In the current study, even though the average size of HDL particles did not vary with an increasing number of freeze/thaw cycles, medium- and large-sized HDL particles appeared to be converted to small HDL particles with an increasing number of cycles. The standard deviation of the number of different sized particles did not increase suggesting that a similar shift in particle size occurred for all particles and comparisons between groups would be relative. Thus, sizing of HDL can be analyzed in stored plasma, as long as samples are all thawed an equal number of times and one is aware that there could be an exaggerated increase in the number of smaller HDL particles. Though the sizes differed, there was no effect of freeze/thaw on the amount of peptide counts for each protein carried by HDL, including the apolipoproteins. We also determined the impact of freezing and thawing on LDL and VLDL sizing. VLDL sizing did not change with freezing and thawing of samples. There was a decrease in the number of large LDL and an increase in the number of IDL, however. As the internal non polar core of the particle would need to be increased by lipids for this to occur, it is possible that either some of the core triglyceride or cholesteryl ester from medium and large HDL blebs off and combines with the large LDL to form IDL or LDL particles fuse together to form IDL. Unlike HDL, the standard deviation for large LDL increases with increasing number of thaws suggesting that not all particles change the same with thawing in the cold. Thus, it appears that as long as samples have been thawed three times, sizing can be performed. More than three thaws would suggest potential misrepresentation of the number of large LDL particles. As LDL heterogeneity is the cause of various functions of different sized LDL and IDL particles [25, 26], it is important to know the number of thaws before interpreting data obtained.

Cytokine concentrations were also measured and do not appear to be as constant as being lipoprotein-cholesterol and triglyceride concentrations when samples are repeatedly thawed and refrozen. Previous studies have shown marked differences in responses from very little effect of freezing and thawing on cytokine and CRP measurements to dramatic effects [27–30]. Using the newer Multiplex methodology, we found that IL-6, IL-1β, and hsCRP concentrations decreased as the number of thaws increased, whereas IL-8 and TNFα concentrations did not vary significantly, using 20% day-to-day variability as our cut off for physiological changes [28– 30]. Importantly, the most marked decreases in concentration after multiple freeze/thaw cycles were those in samples with markedly elevated concentrations. When averages were recalculated after removing the samples with the greatest measured values, thawing and refreezing samples had reduced effects on cytokine concentrations.

In summary, we have found that there are statistically significant decreases in circulating levels of lipoprotein-cholesterol, triglyceride, and cytokines with multiple freeze thaw cycles and with long term storage; one limitation of this study was that the long term storage was only 18 months. For the lipids, though significant, changes were relatively small and would be less than that which would occur from day-to-day variation. TNF α and IL-8 had relatively small reductions with multiple freeze/thaw cycles, whereas IL-6, IL-1β, and hsCRP had more significant reductions in measured concentrations by the seventh thaw. Interestingly, the greatest reductions in measured concentrations in hsCRP and plasma cytokines occurred in samples with greatly elevated concentrations. The greatest impact of repeated thawing and refreezing of samples was in a shift of HDL particle size from larger to smaller particles, without a change in the HDL proteome, and a shift of large LDL to IDL. As standard deviations do not increase with the number of thaws for HDL and IDL, samples should all change relatively similarly. However, the effect of repeated freeze/ thaw cycles did lead to an increase in the standard deviation of large LDL particles such that particle number could be underestimated in some groups as changes were not consistent in all samples. Therefore, as long as all samples are handled similarly and one knows the number of times samples have been previously thawed, re-assaying samples from previous studies cytokine concentrations and lipoprotein composition, is appropriate and will aide in defining new biomarkers more rapidly and efficiently.

## Figures and Tables

**Table 1:** Plasma levels of lipids, cytokines, hsCRP, and apoA-I after thawing samples multliple times in the cold or at room temperature and after long-term storage.

Thaw								
Measurement	1	2	3	4	7	1	3	1
(COLD)						(RT)		(18 mos)
**Lipid (mg/dl)**								
LDL-C	94.3 ± 29.0	93.7 ± 29.3	93.2 ± 29.1*	92.9 ± 29.0*	90.8 ± 27.8*	93.9 ± 29.1	93.3 ± 28.9	93.8 ± 29.7
HDL-C	61.1 ± 18.2	60.5 ± 18.4*	60.1 ± 18.3*	59.9 ± 18.3*	58.9 ± 17.9*	60.3 ± 18.1	59.9 ± 18.1	64.7 ± 19.4
TC	178.5 ± 38.0	178.2 ± 38.6	176.8 ± 38.1*	176.8 ± 37.7*	174.1 ± 36.6*	177.7 ± 38.0	177.1 ± 38.2	170.8 ± 35.8
TG	91.9 ± 43.2	91.8 ± 41.5	90.0 ± 39.3	88.5 ± 39.0*	83.4 ± 34.4*	91.5 ± 42.6	88.6 ± 39.4	84.7 ± 37.9
**Cytokines (pg/ml)**								
IL-6	2.7 ± 3.8	2.3 ± 3.2*	2.5 ± 5.0*	2.4 ± 3.1*	2.0 ± 2.5*Ψ	2.6 ± 4.2*	2.5 ± 2.9*	2.0 ± 3.8*
TNFα	14.8 ± 14.3	13.3 ± 9.5*	12.8 ± 9.3*	13.7 ± 10.6	13.1 ± 10.7*	13.9 ± 14.7	13.6 ± 12.7	12.3 ± 4.3
IL-8	7.4 ± 8.0	6.8 ± 5.7	6.8 ± 6.1*	7.1 ± 65.9	6.8 ± 5.4*	7.0 ± 6.5	7.2 ± 5.9	9.8 ± 13.4
IL-1β	4.6 ± 17.8	4.3 ± 19.4	3.2 ± 11.5*	3.9 ± 15.7*	3.6 ± 15.5*Ψ	4.4 ± 19.8	7.5 ± 41.9	2.1 ± 7.4*
hsCRP (mg/L)	7.1 ± 10.6	6.4 ± 9.4*	6.1 ± 8.4*	6.1 ± 8.5*	5.6 ± 7.9*Ψ	5.5 ± 6.9*	4.7 ± 6.4*	10.7 ± 14.5*
apoA-I (mg/ml)	1.5 ± 0.6	1.3 ± 0.5*	1.3 ± 0.8	1.3 ± 0.6*	1.3 ± 0.7*	1.4 ± 0.9*	1.5 ± 0.9	2.1 ± 0.7*

Data are presented as means ± SD for 51 individual participants.

* represents statistically significant differences from the first thaw (P<0.05).

Ψ represents values that are physiologically different from the first thaw based on previously reported day-to-day variations of lipid, cytokine, and hsCRP concentrations. Comparisons were made between cold thaws, between the first cold thaw and the thaws at room temperature and between the first cold thaw and the thaw of the sample stored for 18 months.

**Table 2:** The number and size of lipoprotein particles after multiple freeze thaw cycles in the cold or at room temperature and after long-term storage.

Thaw								
Measurement	1	2	3	4	7	1	3	1
(COLD)						(RT)		(18 mos)
**Lipoprotein –NMR**								
**HDL**								
Particle #	35.4 ± 1.9	35.5 ± 2.0	35.2 ± 2.3	35.0 ± 2.5	33.7 ± 1.3*	34.7 ± 2.1	36.1 ± 2.3	35.0 ± 2.2
Avg size (nm)	10.0 ± 0.2	9.9 ± 0.2	9.9 ± 0.2	9.9 ± 0.2	9.9 ± 0.2	10.0 ± 0.1	9.9 ± 0.2	10.0 ± 0.2
Small HDL (#)	6.9 ± 4.7	9.9 ± 2.8	10.1 ± 3.0*	12.5 ± 4.0*	12.1 ± 3.7*	7.4 ± 5.0	8.2 ± 4.5	10.7 ± 3.2
Medium HDL (#)	18.7 ± 4.1	15.8 ± 3.6	15.4 ± 3.8	12.9 ± 3.7*	12.3 ± 4.2*	17.6 ± 4.9	17.9 ± 4.6	14.8 ± 2.7*
Large HDL (#)	9.6 ± 1.3	9.2 ± 1.6*	9.1 ± 1.7*	9.1 ± 1.8*	8.5 ± 1.6*	9.2 ± 1.5	9.5 ± 1.4	9.1 ± 1.6
**LDL**								
Particle #	834 ± 159	842 ± 191	845 ± 185	845 ± 198	761 ± 140	767 ± 126	817 ± 170	818 ± 166
Avg size (nm)	20.8 ± 0.4	20.9 ± 0.4	20.8 ± 0.2	20.6 ± 0.3	20.8 ± 0.4	20.7 ± 0.3	20.8 ± 0.4	20.8 ± 0.3
Small LDL (#)	248 ± 194	252 ± 240	255 ± 191	288 ± 177	261 ± 162	242 ± 122	207 ± 122	202 ± 179
Large LDL (#)	396 ± 153	393 ± 148	322 ± 72	238 ± 135*	195 ± 124*	285 ± 106	338 ± 136	326 ± 113
Very large LDL (#)	914 ± 148	991 ± 160	983 ± 170	920 ± 184	895 ± 137	864 ± 117	932 ± 139	907 ± 158
IDL (#)	116 ± 55	122 ± 95	182 ± 84	234 ± 127*	228 ± 57*	163 ± 47	200 ± 81	222 ± 87*
**VLDL**								
Particle #	41.0 ± 7.3	48.1 ± 6.1	42.0 ± 9.2	40.1 ± 11.9	37.7 ± 10.6	37.9 ± 8.1	41.6 ± 4.1	40.5 ± 11.0
Avg size (nm)	49.1 ± 2.1	47.2 ± 1.7	48.7 ± 2.1	50.1 ± 2.3	50.5 ± 2.2	50.6 ± 2.2	49.1 ± 1.4	49.8 ± 1.8
Small VLDL (#)	27.1 ± 9.0	33.7 ± 3.3	26.2 ± 9.1	26.8 ± 8.4	28.7 ± 8.4	23.4 ± 3.2	28.6 ± 4.4	24.7 ± 6.5
Medium VLDL (#)	14.4 ± 9.2	14.5 ± 5.3	16.3 ± 5.1	13.8 ± 4.2	9.6 ± 2.0	15.4 ± 5.0	13.2 ± 3.4	14.2 ± 4.5
Large VLDL (#)	2.7 ± 0.4	3.0 ± 1.2	2.9 ± 0.8	2.8 ± 0.6	2.7 ± 0.8	3.0 ± 1.0	3.1 ± 1.1	2.7 ± 0.8

Data are presented as means ± SD for 6 pooled samples.

* represents significant differences from the first cold thaw (P<0.05). Comparisons were made between cold thaws, between the first cold thaw and the thaws at room temperature and between the first cold thaw and the thaw of the sample stored for 18 months.

**Table 3:** Peptide counts of different proteins of the HDL proteome after several thaws in the cold and at room temperature Proteins presented are those with no change in peptide counts, a decrease in peptide counts, and the apolipoproteins with the highest peptide counts. Of the remaining 32 proteins analyzed, none changed with freeze/thaw cycles.

	Thaw						
Protein	1	2	3	4	7	1	3
(COLD)						(RT)	
Complement C3	43 ± 8	43 ± 7	43 ± 10	42 ± 12	45 ± 6	40 ± 11	40 ± 11
Vitamin D BP	11 ± 3	10 ± 4	10 ± 4	12 ± 3	11 ± 3	11 ± 4	11 ± 4
Fibrinogen α chain	10 ± 2	8 ± 1	8 ± 2	8 ± 2	6 ± 2*	8 ± 2	8 ± 2
Fibrinogen β chain	9 ± 1	8 ± 2	7 ± 2	7 ± 2	5 ± 2*	6 ± 2	7 ± 1
apoA-I	32 ± 5	29 ± 7	27 ± 9	30 ± 5	30 ± 9	31 ± 2	31 ± 3
apoA-IV	12 ± 3	12 ± 2	11 ± 3	11 ± 2	10 ± 3	9 ± 4	12 ± 1
apoA-II	7 ± 1	7 ± 2	7 ± 1	7 ± 2	7 ± 1	8 ± 1	7 ± 1
apoC-III	2 ± 0	2 ± 0	2 ± 1	2 ± 0	2 ± 1	2 ± 0	2 ± 0

Data are presented as means ± SD.

* represents significant differences between multiple thaws and the first thaw in the cold (P<0.05) not correcting for the multiple number of comparisons. No proteins were different from the first thaw in the cold when corrected for the number of proteins compared.
